# The *R337H *mutation in *TP53 *and breast cancer in Brazil

**DOI:** 10.1186/1897-4287-10-3

**Published:** 2012-03-28

**Authors:** Magda CB Gomes, Joanne Kotsopoulos, Gutemberg Leão de Almeida, Mauricio M Costa, Roberto Vieira, Firmino de AG Filho, Marcos B Pitombo, Paulo Roberto F Leal, Robert Royer, Phil Zhang, Steven A Narod

**Affiliations:** 1 Department of Oncology, Antonio Pedro University Hospital, Fluminense Federal University, Rio de Janeiro, Brazil; 2 Department of Internal Medicine, Pedro Ernesto University Hospital, State University of Rio de Janeiro, Rio de Janeiro, Brazil; 3 Women's College Research Institute, Women's College Hospital, Toronto, ON, Canada; 4 Department of Gynecology and Obstetrics, Institute of Gynecology, Federal University of Rio de Janeiro, Rio de Janeiro, Brazil; 5 Department of Gynecology and Obstetrics, Clementino Fraga Filho University Hospital, Federal University of Rio de Janeiro, Rio de Janeiro, Brazil; 6 Instituto Fernandes Figueiras, Rio de Janeiro, Brazil; 7 The Centre of Tumor Treatment, Botafogo, Rio de Janeiro, Brazil; 8 Department of Surgery, Pedro Ernesto University Hospital, Rio de Janeiro State University, Rio de Janeiro, Brazil; 9 Women's College Research Institute, 790 Bay Street, Room 750, Toronto, Ontario M5G 1N8, Canada

**Keywords:** Brazil, Breast cancer, *TP53 *mutation, Adrenal cortical carcinomas

## Abstract

**Background:**

Germline mutations in p53 are associated with the Li-Fraumeni Syndrome which is characterized by childhood cancers, including pediatric adrenal cortical carcinomas and early onset breast cancer. The high incidence of adrenal cortical carcinomas in southern Brazil is mostly attributed to the *R337H *mutation in *TP53*. The relatively high population frequency of this mutation in southern Brazil, along with the clustering of early onset breast cancer in Li-Frameni families, suggests this mutation may also be a low-penetrance breast cancer susceptibility polymorphism.

**Methods:**

We undertook this study to evaluate the frequency of the *R337H *mutation in breast cancer patients from Rio de Janeiro, Brazil. *R337H *mutation status was determined in 390 unselected breast cases and 324 controls identified from clinics in Rio de Janeiro, Brazil using a PCR-based assay.

**Results:**

Two of the breast cancer cases (0.5%) and none of the controls carried the mutation. Both cases had an early age at diagnosis (< 40 years old) and a family history of breast and other cancers.

**Conclusions:**

These data suggest genetic screening of young onset breast cancer patients should include testing for the *R337H *mutation.

## Introduction

Germline mutations in p53 are associated with Li-Fraumeni Syndrome which is characterized by childhood cancers and early onset breast cancer [1]. The spectrum of tumors includes soft tissue sarcomas, osteosarcomas, breast cancers and pediatric adrenal cortical carcinomas. It has been reported that the incidence of pediatric adrenal cortical carcinomas in southern Brazil is 10-15 times higher that the incidence in the United States [2]. Studies by Ribeiro *et al. *have shown that the majority of adrenal cortical carcinoma cases in southern Brazil are attributed to one specific germ-line point mutation in exon 10 at codon 337 (CGC → CAC) of *TP53 *resulting in an arginine-to-histidine amino acid substitution (*R337H*) [3].

Initially, it was believed that this mutation did not confer an increased risk of developing additional cancers [4]; however, various groups have suggested that this mutation also increases the risk of breast cancer in women living in southern Brazil [5-8]. Given that Li-Fraumeni syndrome patients display higher than normal rates of breast cancer, we undertook this study to evaluate the frequency of the *R337H *mutation in a case-control study of breast cancer conducted in Rio de Janeiro, Brazil.

## Methods

### Study design

The study was conducted in Rio de Janeiro, Brazil between February 2003 and September 2005. In Brazil, patients with breast cancer are treated for in both public and private settings. To ensure that patients from a wide range of ethnic and social backgrounds were included in the current study, patients were recruited from both a public hospital (Hospital of the Federal University of Rio de Janeiro) and from two private medical clinics (The Centre of Tumor Treatment, Botafogo, Rio de Janeiro, Brazil and the clinic of Roberto Vieira in Copacabana, Rio de Janeiro) all of which provide oncology services. Protocols were approved by the institutional review boards at all the participating centers.

### Study population

Potential case subjects were approached to participate in the study during an out-patient visit to the medical oncology clinic, or during hospital admission. Cases were women between 20 and 60 years of age and who had been diagnosed with invasive breast cancer between 1978 and 2005. In total, 382 women were approached to participate, of whom 381 agreed (one women declined because she did not wish to receive genetic testing). There were 195 patients recruited from the public hospitals and 186 patients recruited from the private clinics. Three cases had a past history of other cancers (two lymphomas and one melanoma). Potential control subjects were women without a diagnosis of breast cancer, but who sought medical attention for another condition. Controls were derived from the same public hospital and medical clinics and as the cases. All controls were in-patients in medical and surgical wards and were being treated for a range of non-cancerous conditions. In total, 486 controls were recruited (no potential control declined). All women provided written informed consent to participate in the study.

### Data and sample collection

All of the study subjects participated in a structured interview that collected information about reproductive, hormonal, and lifestyle exposures. Women were requested to provide a sample of blood for genetic testing and to provide details about their family history of cancer.

### Mutation analysis

DNA was extracted from peripheral blood leukocytes using Puregene DNA Isolation Kit (Gentra Systems, Minneapolis, USA). Mutation analysis was performed on 390 cases and 324 controls using the tetra-primer amplification refractory mutation system (ARMS) [9]. Two allele-specific amplicons are generated using two pairs of primers. One pair produces an amplicon that represents the wildtype allele, and the other corresponding to the mutant allele. These two amplicons differ in length, which allowing for discrimination by agarose gel electrophoresis. All mutations detected were confirmed by direct sequencing [BigDye Terminator v.3.1 Cycle Sequencing Kit, and 3130XL Genetic Analyzer (Applied BioSystems)] according to manufacturer's instructions. Primers used for assay and sequencing are available upon request.

## Results

**Figure 1:**
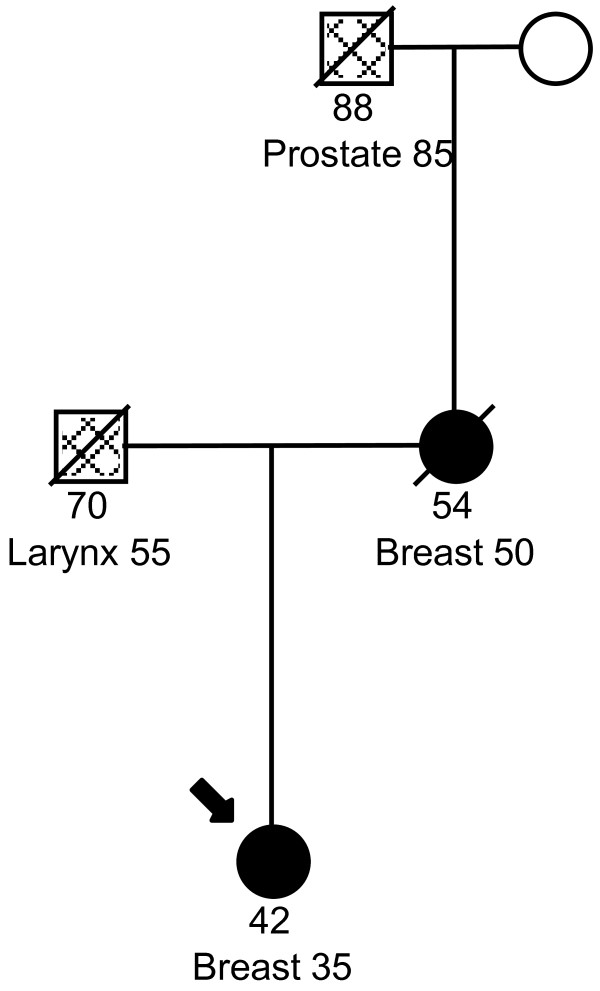
**Pedigree for breast cancer case diagnosed at age 35 with a *TP53 R337H*mutation**.

**Figure 2:**
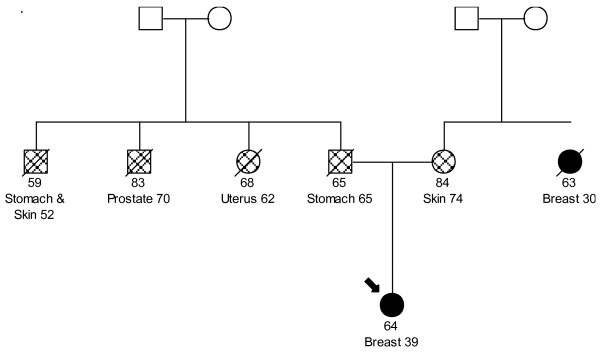
**Pedigree for breast cancer case diagnosed at age 39 with a *TP53 R337H*mutation**.

DNA from a total of 716 study subjects was tested for the *R337H *mutation in *TP53*. The mean age at diagnosis of cases was 46 years old (range 25-66) and the mean age at interview of the controls was 40 years old (range 19-63). There were 87 cases that were diagnosed at or before age 40. Of the 390 cases tested, we identified two cases with a *R337H *mutation (mutation frequency 0.5%). Pedigrees for the two cases are displayed in Figure 1 and 2. None of the 324 controls were found to carry this mutation. The prevalence of mutations among women ≤ 40 years of age was 2.3%. The ages of diagnoses for the two cases with the *R337H *mutation were 35 and 39. The first breast cancer case with a *R337H *mutation was diagnosed with breast cancer at age 35. Her mother was diagnosed with breast cancer at age 50 and her father had a diagnosis of laryngeal cancer at age 55. Her maternal grandfather had a diagnosis of prostate cancer at age 85. The second breast cancer case was diagnosed at age 39 with bilateral breast cancer. Her mother had a diagnosis of nonmelanoma skin cancer at age 74 and her maternal aunt had a diagnosis of breast cancer at age 30. Her father and paternal uncle were diagnosed with gastric cancer at age 65 and 52, respectively while a different paternal uncle developed prostate cancer at age 70. A paternal aunt was diagnosed of uterine cancer at age 62.

## Discussion

Our study suggests that the prevalence of the *TP53 R337H *mutation in breast cancer patients diagnosed in clinics of Rio de Janeiro, Brazil is low. Only two of the 390 cases were found to carry this mutation (~0.5%) with an age of diagnosis of 35 and 39 (bilateral breast cancer). This estimate is substantially lower than that obtained in previous studies conducted in southern Brazil (range 2.4-11%) which included study participants from Rio de Janeiro, Sao Paolo and Porto Alegre [5,8]. Sao Paolo and Porto Alegre are 442 km and 1546 km from Rio de Janeiro, respectively. They did not evaluate frequency based on city; however, all three cities are located on the eastern side of Brazil and close to the Atlantic ocean. Despite similarities in geographic regions and the mutation of interest, these studies were substantially different with respect to the selection of their study populations and may explain why our mutation frequency was substantially lower. For example, the current study included unselected cases of breast cancer, while the two other studies included subjects with families suggestive of Li-Fraumeni syndrome [5,8].

Achatz *et al. *screened 45 Brazilian subjects from unrelated families with cancer histories suggestive of Li-Fraumeni syndrome and 53 healthy subjects for the *R337H *mutation [8]. They found a *TP53 *mutation in 13 cases, six of which were the *R337H *mutation. None of the 53 healthy subjects carried the mutation. In addition, the most common type of tumor in these families was that of the breast (~30.4%) [3]. In additional studies, the same researchers identified the *R337H *mutation in two out of 750 healthy women (0.3%) enrolled in a breast screening program in Porto Alegre, southern Brazil [7]. Both cases had a family history of cancer but did not match the Li-Fraumeni syndrome clinical profile.

Assumpcao *et al. *estimated the prevalence of this mutation in 123 breast cancer cases and 223 age-matched controls [5]. Three of the cases (2.4%) and none of the controls were carriers of the *R337H *mutation (*P *= 0.04). All three cases had an early age at diagnosis (range 19-44 years old) and two of the three cases had a family history suggestive of Li-Fraumeni syndrome. Interestingly, all three tumors showed loss of heterozygosity of the mutant allele versus the wild-type allele in contrast to pediatric adrenal cortical carcinomas that normally retain the mutant allele [3,10]. Given that not all families with the *R337H *mutation present with other cancers, the authors tested for the presence of other genetic variants within *TP53 *(R72P and ins16) and *MDM2 *(SNP309) that have been reported to affect tumor susceptibility and age at onset in R337H mutation carriers, respectively [11-14]. Two of the cases were heterozygous both the TP53 SNPs, while the third patient was homozygous for both R72P. Two of the cases also carried the MDM2 polymorphism. Based on these findings, it has been suggested that the R337H mutation in combination with other SNPs may affect p53 function and ultimately, breast cancer predisposition.

Functional analysis has shown the mutant p53 protein to be pH-sensitive and may adopt a mutant phenotype only under particular physiological conditions [3,10]. Further, the authors have shown that the mutated tumor cells demonstrate a loss of heterozygosity of the wild-type allele with retention of the mutant allele and accumulation of mutant p53 protein in the nucleus. The penetrance of adrenal cortical carcinomas among carriers of this mutation is 9.9% (95%CI 8.7-11.1%) by the age of 12 years [4]. The frequency of this mutation in southern Brazil has been estimated to be approximately 0.2%-0.3% [7]. Due to this high population frequency the *R337H *mutation, along with the high incidence of adrenal cortical carcinomas, has led to the implementation of screening all newborns in the state of Parana in southern Brazil [15].

Strengths of the current study include the large sample size and extensive information on family history. All the participants in this study were recruited from public and private clinics in Rio de Janeiro, Brazil and thus our results are generalizeable to population of Brazil.

## Conclusions

We have previously identified a *BRCA1 *or *BRCA2 *mutation in 2.3% of women with breast cancer using the same study population [16]. The frequency of the *BRCA *mutation is substantially higher compared with the *R337H *mutation and thus, may play a more important role in predisposing to breast cancer in this South American population. Nonetheless, given the high population frequency of the *R337H *mutation in southern Brazil, this might have an important implication with respect to genetic screening of breast-cancer predisposing genes. Additional research is needed to determine if this mutation should be added to existing panel of mutations when screening individuals with a family history.

## Abbreviations

BRCA1: Breast cancer susceptibility gene 1; BRCA2: Breast cancer susceptibility gene 2.

## Competing interests

The authors declare that they have no competing interests.

## Authors' contributions

SAN conceived the study. JK participated in the study design, statistical analysis and manuscript preparation. MCBG, GLA, MMC, RV, FAGF, MBP, and PRFL participated in the design of the study and in the supervision of the accrual of study participants. RR and PZ conducted the mutation analysis. All authors read and approved the final manuscript.
